# Spoof Surface Plasmon Polaritons Power Divider with large Isolation

**DOI:** 10.1038/s41598-018-24404-0

**Published:** 2018-04-13

**Authors:** Shiyan Zhou, Jing-Yu Lin, Sai-Wai Wong, Fei Deng, Lei Zhu, Yang Yang, Yejun He, Zhi-Hong Tu

**Affiliations:** 1 School of Electronic and Information Engineering, South China University of Technology, Guangzhou City, Guangdong Province, 510640 China; 2 College of Information Engineering, Shenzhen University, Shenzhen, 518060 China; 3 School of Electrical and Data Engineering, University of Technology Sydney, Ultimo, NSW 2007 Australia; 4 Department of Electrical and Computer Engineering, Faculty of Science and Technology, University of Macau, Macau, SAR 999078 China

## Abstract

Periodic corrugated metal structure is designed to support and propagate spoof surface plasmon polaritons (SSPPs) wave in the microwave frequencies. In this paper, firstly a plasmonic waveguide consisting of oval-ring shaped cells is proposed with the performance of high transmission efficiency in a wide frequency range. The coplanar waveguides (CPWs) with 50 Ω impedance are adopted to feed the energies or extract signals at both ends of the plasmonic waveguide. Then a well-isolated power divider is constructed based on the SSPPs waveguides aiming to equally split the energy of the SSPPs wave into two equal parts. The stepped-impedances are co-designed with the three input/output ports of the power divider to achieve the impedance-matching between the SSPPs waveguides and the coplanar waveguides. Besides, a single resistor is placed in the middle of two symmetrical half oval-rings to realize the isolation between the two output ports over the spectrum of 4.5–7.5 GHz. Finally, both plasmonic waveguide and the power divider are fabricated and tested to verify the predicted characteristics.

## Introduction

Surface plasmon polaritons (SPPs) are a kind of electromagnetic waves generated by the interaction between the light and free electron on the metal surfaces. Due to the highly localized characteristics of the SPPs wave in sub-wavelength scale between the metal and dielectric, it turns out to be a promising solution by using the SPPs for loss reduction in microwave and terahertz (THz) frequencies. However, at the low-frequency band, the SPPs wave no longer propagates along the metal due to the changed metal dielectric constant. In recent years, it has been found that the corrugated surfaces waveguide can be implemented by constructing the structures of periodic hole cells^1–3^, which excites the TM mode of the SSPPs similar to the SPPs in optics. In order to design and apply SSPPs waveguides and devices in microwave low-frequency band, it is necessary to connect the new SSPPs waveguide to the conventional waveguide with a matching conversion. The SSPPs waveguides connected to microstrip lines^4,5^, coplanar waveguides (CPW)^6,7^ or slot^8,9^ are proposed to adapt to different circuit environments, which show the features of broad frequency bands and the controllable cut-off frequencies. With the development of the SSPPs devices in microwave and THz bands, various SSPPs filters^10–13^ and antennas^14–17^ have been studied.

As one of the key passive components in microwave circuits and communication systems, power divider divides the power of the input signal into two output channels with equal or unequal power levels. The research on how to divide SPPs wave has been reported, which uses the Y-splitter construction in optics^18^. For the microwave bands, the SSPPs splitters^19,20^ are also studied to achieve the division of two or more different frequencies. A multiway power divider^21^ is proposed by the theory of the transformation optics, which demonstrates the reflection influence due to the bent section of the branches of the power divider. Moreover, a SSPPs power divider of Y structure^22^ is presented. However, a good isolation between/among the outputs in those reported power dividers or splitters has yet been achieved. Wilkinson splitter has the inherent nature of good isolation between/among the output channels, however, the working frequency band of the conventional Wilkinson splitter is very narrow, and only the band located at the center frequency can achieve good matching and high isolation performance. Fortunately, the recently reported SSPPs structures indicate the great potentials to deal with the challenges of a standard Wilkinson power divider.

In this paper, we firstly present a broadband SSPPs inspired Wilkinson power divider with oval-ring periodic structures. High-efficiency conversion is achieved between the plasmonic waveguide and the coplanar waveguide in a wide frequency range. The designed SSPPs waveguide is split into two halves along the symmetrical axis. A pair of stepped-impedance structures are co-designed in the input/output channels in order to match the impedance between the CPW and the new half oval-ring shaped SSPPs waveguide. Moreover, a power divider based on two half oval-ring SSPPs waveguide is proposed. A single resistor is engineered between the two symmetrical half oval-rings to achieve high-isolation between the output channels. The proposed power divider has the great potentials in the applications of the plasmonic integrated circuits and systems at the microwave frequency bands.

## Results

### SSPPs waveguide

**Figure 1:**
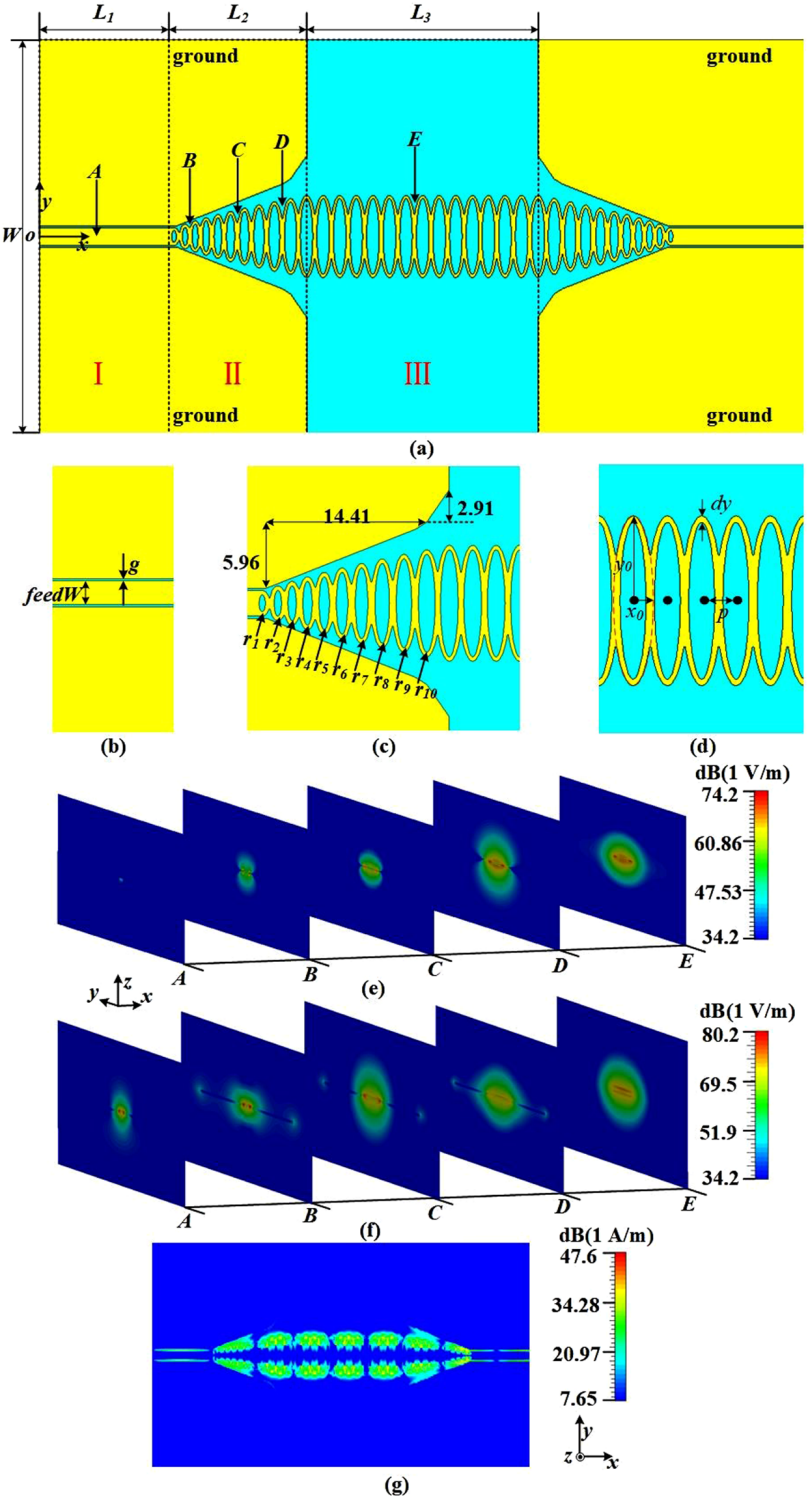
The configuration of the SSPPs waveguide with three divided sections. (**a**) The combination of CPW and SSPPs, in which *L*_1_ = 16.3 mm, *L*_2_ = 17.5 mm, *L*_3_ = 29.4 mm, and *W* = 50 mm. (**b**) The CPW section, in which *g* = 0.2 mm, *feedW* = 2.3 mm. (**c**) The match section with ten oval-rings of gradient scale and flaring ground. (**d**) The corrugated metal strip composed of oval-ring shaped periodic structures, in which the ellipse long axis radius *y*_0_ = 5.2 mm, the ellipse short axis radius *x*_0_ = 1.2 mm, the width of the oval-ring *dy* = 0.4 mm, and the distance between the two adjacent oval-ring centers *p* = 2.1 mm. (**e**) The electrical amplitude distributions of 5 marked planes along the *x*-axis direction. (**f**) The electrical amplitude distributions of 5 marked planes perpendicular to the *x*-axis direction. (**g**) The magnetic distribution of oval-ring SSPPs waveguide at 8 GHz in *x-y* plane.

As shown in Fig. 1(a), the oval-ring shaped SSPPs waveguide consists of three sections. The first section I is CPWs with 50 Ω impedance (shown in Fig. 1(b)), as the feeding part of the input or output. Section II is designed to make smooth conversion between the CPW and the plasmonic waveguides. The oval-rings of the gradient scale and flaring ground are presented to match the impedance between the CPW and SSPPs waveguides. Figure 1(c) and Table 1 depict the details of the transition section. The width of the different oval-rings is assigned to be 0.4 mm. In addition, the curve of the flaring ground is divided to two straight lines. One is designed from the first oval-ring to the tenth oval-ring along the profile of these oval-rings of gradient scale, the other starts from the tenth oval-ring of the section II and ends in the first oval-ring of section III. The third section III is SSPPs waveguide constructed by several identical oval-rings connected to each other. The designed periodic parameters of the proposed structure are shown in Fig. 1(d). The distance between the centers of two adjacent oval-rings is smaller than the short axis radius of the ellipse which is marked with a red dotted line in Fig. 1(d), so the grooves on the sides of the oval-rings’ strip are formed. As the depth of the groove increases, the cut-off frequency becomes lower. Therefore, the cut-off frequency can be controlled effectively by changing the distance between the two adjacent ellipses. Based on the SSPPs wave spreading along these grooves and highly localized in metal surfaces, the oval holes in the ellipse centers is adopted to design the SSPPs waveguide of oval-ring shaped structures.

**Table 1:** *x*_*n*_ is the short axis radius of the *n*th oval-ring, *y*_*n*_ is the long axis radius of the *n*th oval-ring, *p*_*n*_ is the distance between the *n*th and the *n* + *1*th oval-ring centers.

R_n_	R_1_	R_2_	R_3_	R_4_	R_5_	R_6_	R_7_	R_8_	R_9_	R_10_
*x*_*n*_	0.7	0.75	0.8	0.85	0.9	0.95	1	1.05	1.1	1.1592
*y*_*n*_	1.15	1.555	1.96	2.365	2.77	3.175	3.58	3.985	4.39	4.795
*P*_*n*_	1.4	1.28	1.38	1.5	1.6	1.75	1.85	1.95	2.05	2.0184

The CPW section supports the conventional wave of the quasi-TEM mode while the TM mode wave propagates through the SSPPs waveguide. To implement the conversion process between the CPW and SSPPs waveguide in section II, there is a mode conversion process at 5 planes vertical to the *x*-axis, which are marked in Fig. 1(a) from *A* to *E*. Figure 1(e) shows that the electric-field component in *x*-axis direction gradually increases from almost zero to become the major electric-field component of SSPPs waveguide. This component becomes the major electrical field at the SSPPs waveguide. Besides, Fig. 1(f) depicts the varied amplitude distributions of the component perpendicular to the *x*-axis direction. Looking closely at the electric field distribution, the electric field density at the center part is decreasing from A to D. Since this component is the major part of the electric field of the CPW. The electric-field component perpendicular to the *x*-axis direction gradually decreases via the mode converter region (B, C, D) and those reduced energy transforms into the electric-field component in x-axis direction. The electric field distributions in Fig. 1(e) and (f) show that the energy is well confined within the SSPP waveguide region. It can be confirmed that the section II of the oval-rings of gradient scale and flaring ground between CPW and SSPPs waveguide achieves a smooth matching transition from quasi-TEM modes to TM modes. Meanwhile, the magnetic amplitude distribution at 8 GHz is shown in Fig. 1(g). The magnetic field is densely distributed on both sides of the oval-ring units with the center close to zero, which implies the feasibility to simply split the oval-ring waveguide into two halves.

**Figure 2:**
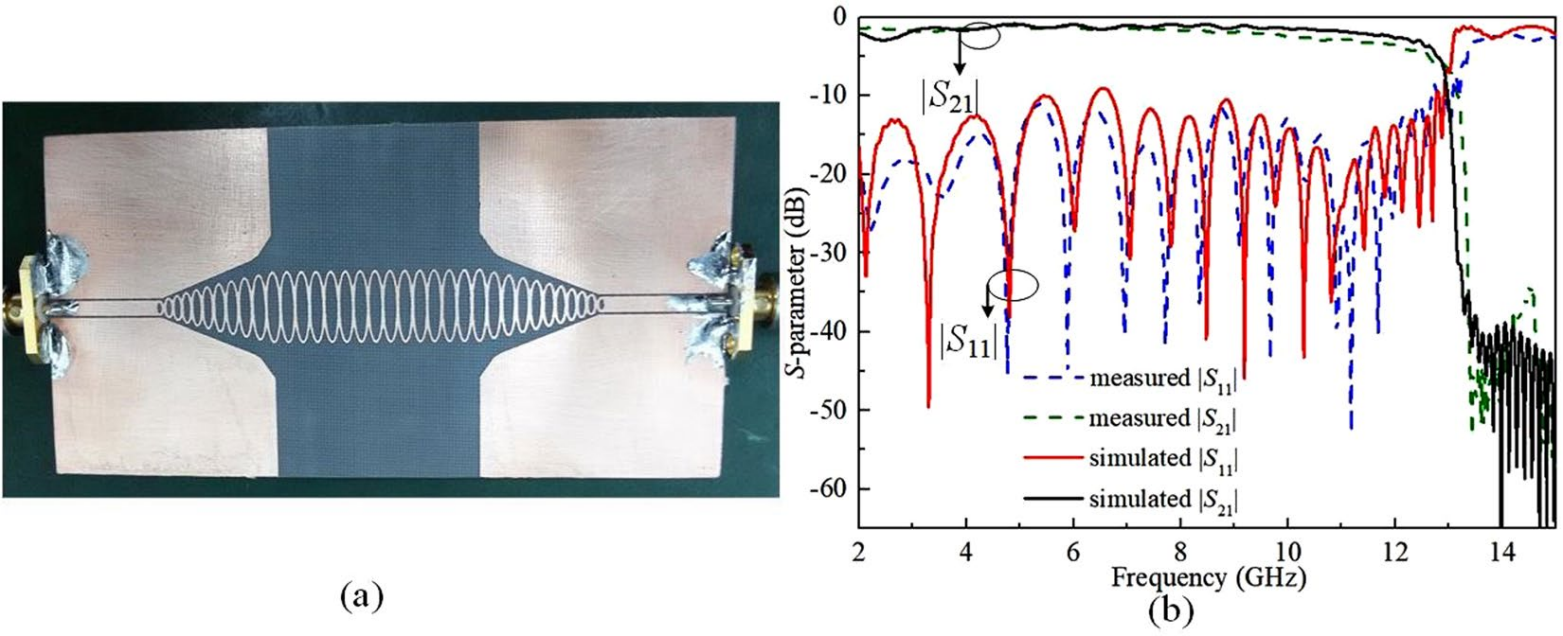
**(a**) The photograph of SSPPs waveguide. (**b**) The simulated and measured *S*-parameter curves of the proposed oval-ring SSPPs waveguide.

Moreover, an experiment is conducted to verify the transmission efficiency of the designed waveguide in Fig. 2(a). As shown in Fig. 2(b), the simulated and measured results are in good agreement. It is noted that the return loss |*S*_11_*|* is almost lower than 10 dB and the insertion loss denoted as |*S*_21_*|* is better than 1.5 dB from 4.3 to 10 GHz, as demonstrated in the solid lines. There are some errors caused during the fabrication processing and the hand soldering of two SMA connectors. The difference of the simulation and measurement in |*S*_21_*|* is less than 0.5 dB from 2 to 8 GHz. As for higher frequency band, the bigger difference is caused mainly by the greater loss of the PCB board in higher frequency band.

### Half oval-ring SSPPs waveguide

**Figure 3:**
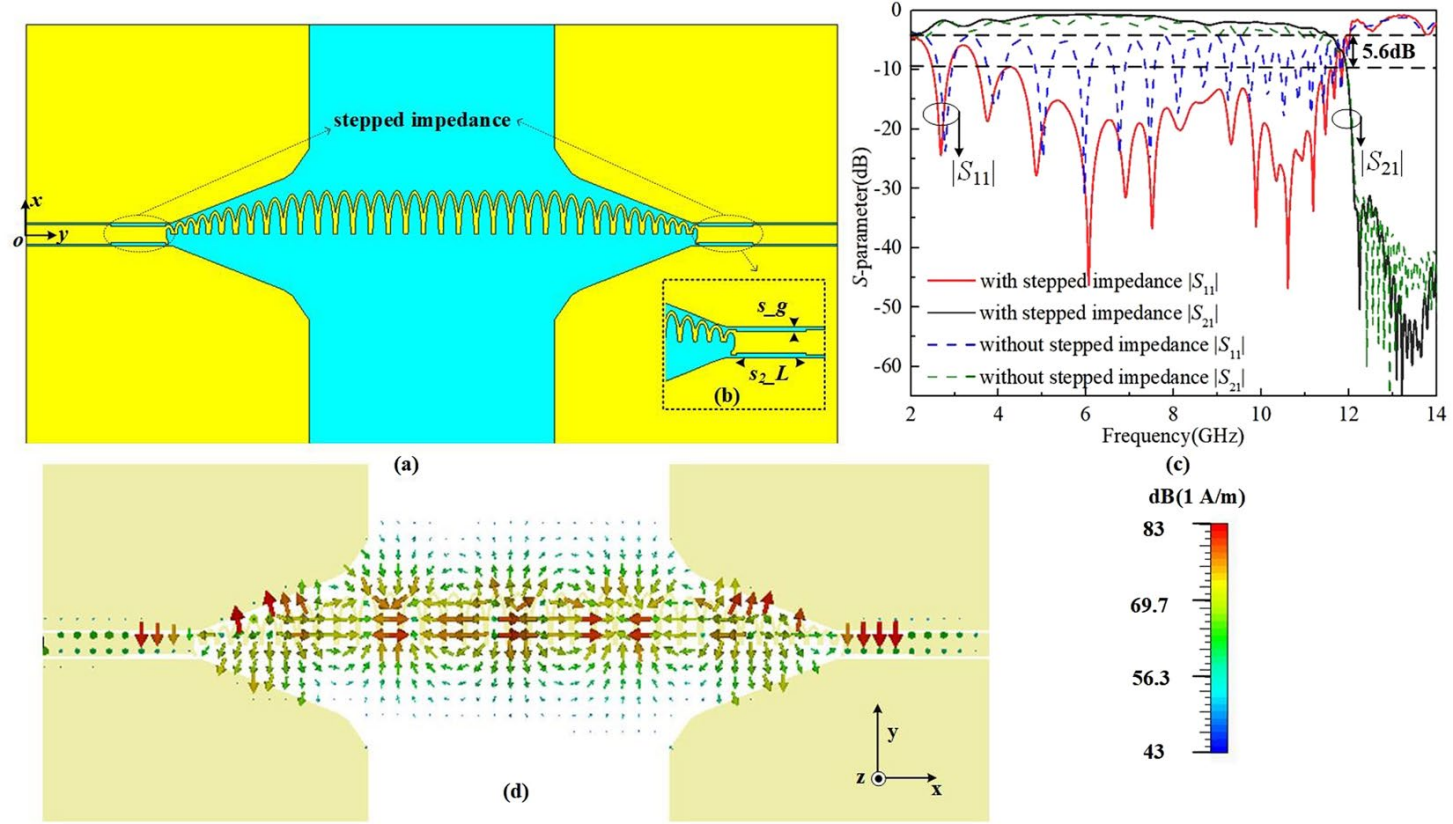
(**a**) The half oval-ring SSPPs waveguide with a pair of stepped impedances. (**b**) The designed stepped impedance parameter, in which *s_g* = 0.2 mm. (**c**) The simulated |*S*_11_| and |*S*_21_| magnitudes of the proposed half oval-ring SSPPs waveguide with different lengths of stepped impedances. (**d**) The electric field distributions at 6 GHz.

In order to get a single band SSPPs waveguide, we equally split sections II and III of the designed SSPPs waveguide into two parts along the *x*-axis. The parameters of the half oval-ring SSPPs waveguide remain unchanged. A half oval-ring SSPPs waveguide is presented in Fig. 3(a). However, the impedance matching condition is changed after a half of oval-ring is cut, thus, the propagation performance is poor. For this reason, a pair of stepped-impedance sections is introduced to match the impedance between the CPW and the half oval-ring SSPPs waveguide, which is described in Fig. 3(b). Figure 3(c) demonstrates the effect of the stepped impedance length *s*_2_*_L* on transmission. As the length increases, the reflection loss denoted as |*S*_11_| decreases. Compared to the length of 0 mm, there is an approximate decrease of 5.6 dB of |*S*_11_| from 4.4 to 7.9 GHz when *s*_2_*_L* = 6.5 mm. The |*S*_21_| with the length of 6.5 mm in the same band remains better than 1.5 dB, which demonstrates an effective wave propagation. Moreover, the electric field distribution of the half oval-ring SSPPs waveguide in the *xoy* plane at 6 GHz is shown in Fig. 3(d). There is the conversion process from the quasi-TEM mode electric field of the CPW to the TM mode of the designed half oval-ring SSPPs.

**Figure 4:**
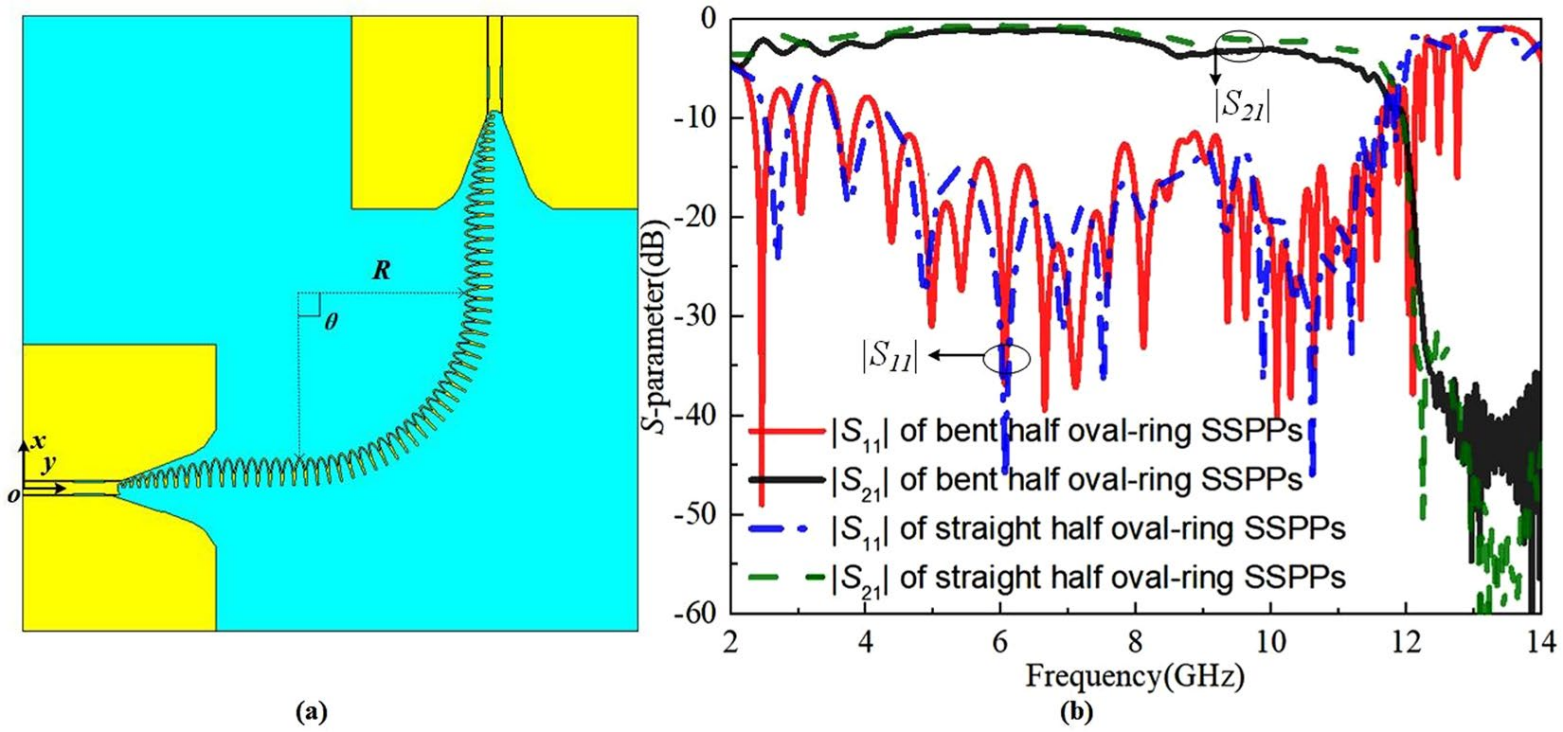
(**a**) The bent half oval-ring SSPPs waveguide, in which *θ* = 90°, *R* = 34 mm and *s2_L* = 5.5 mm. (**b**) The simulated |*S*_11_| and |*S*_21_| magnitudes of the bent half oval-ring SSPPs waveguide.

To demonstrate the effect of bending on the performance of the proposed half oval-ring SSPPs waveguide, the proposed half oval-ring SSPPs waveguide is added a bent circuit with 25 periodic half oval-ring structures along a quarter circle with the radius of *R* = 34 mm, which is described in Fig. 4(a). The simulated results for this bent half oval-ring SSPP waveguide are presented in Fig. 4(b). Compared to straight half oval-ring SSPPs waveguide, there is a bend loss of 0.5 dB of |*S*_21_| from 4 to 8 GHz in bent SSPPs waveguide.

### SSPPs power divider

**Figure 5:**
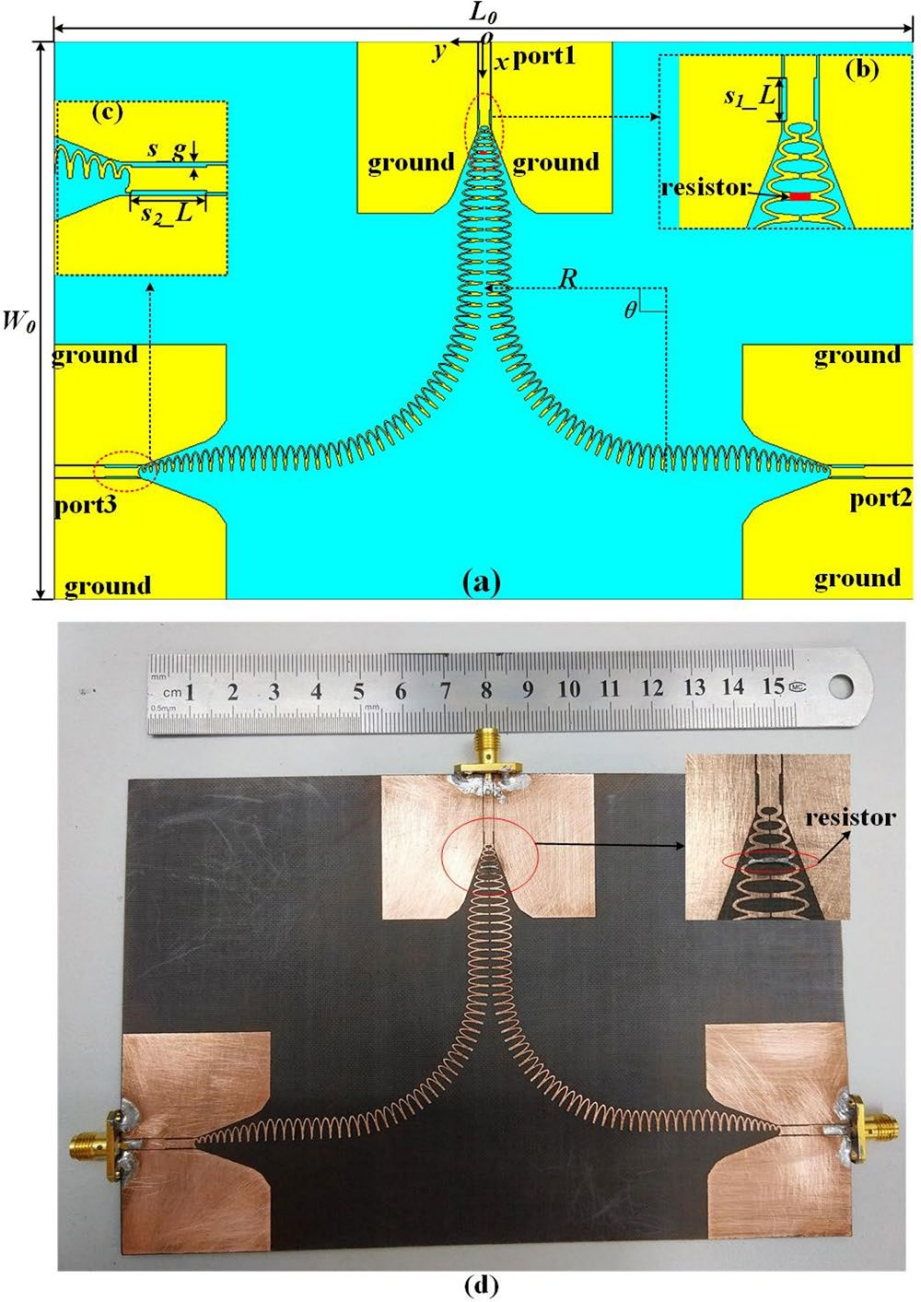
The configuration of the proposed power divider. (**a**) The schematic of power divider, in which *L*_0_ = 169 mm, *W*_0_ = 109.5 mm, the rotation angle of half oval-rings *θ* = 90°, and *R* = 36 mm. (**b**) The stepped impedance of port 2, in which *s*_2_*_L* = 6.5 mm and *s_g* = 0.2 mm. (**c**) The stepped impedance of port 1, in which *s1_L* = 3 mm. And the small red rectangle in the middle between the fourth half oval-ring structures is a 200 Ω resistor. (**d**) Photograph of the power divider. The detail part with a chip resistor is present.

A power divider in Fig. 5(a) is constructed by two identical bent half oval-ring SSPPs waveguides. The port 1 connected the completed oval-ring SSPPs waveguide feeds energy into the structure and the energy is split equally along the directions of two symmetrical bent SSPPs waveguides. Finally, energy can be extracted in the port 2 and port 3, respectively. To reduce the reflection loss, one bent waveguide part in the designed divider consists of 25 periodic half oval-ring structures along a quarter circle with the radius *R* = 36 mm. Unlike [22], where the long match sections consist of the flaring ground, the gradient grooves and taper CPWs and the structure parameters in three ports are different, the proposed design in this work simplifies the conversion impedance-matching area. As the length of the stepped-impedance sections can control the impedance matching, Fig. 5(b) and (c) show the designed parameters of stepped-impedance sections in the input port and the output ports. The depths of these stepped-impedance sections are identical to simplify the design.

As is shown in Fig. 1(f), the magnetic field distribution of SSPPs wave is highly localized in the grooves of the SSPPs waveguide, leading to a weakened electromagnetic energy in the middle of the oval-ring structures. Thus, a narrow gap in the middle of the oval-ring SSPPs waveguide is adopted to add the isolated resistance. Figure 5(d) shows that a 200 Ω resistor is installed in the 0.1 mm gap between the fourth half oval-ring structures in order to achieve high-isolation between port 2 and port 3.

Two-way equal Wilkinson power divider benefits from quarter wave transformers to achieve high isolation. The quarter-wavelength transmission line means there is 90 degrees of the phase change, in which the isolation is achieved by adding an isolation resistor.

**Figure 6:**
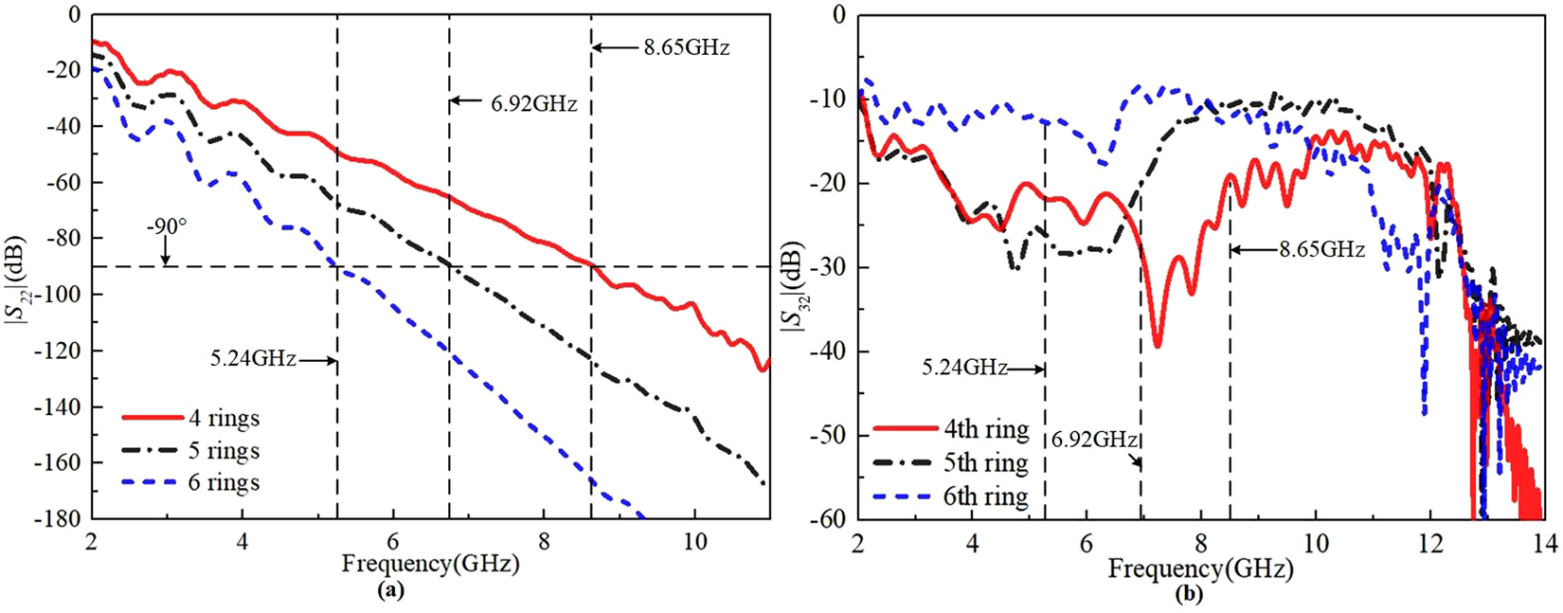
(**a**) The phase of 4th, 5th, and 6th half oval-ring transmission line. (**b**) The comparison of |*S*_32_| by adding a 200 Ω resistor across different half oval-ring structures.

As shown in Fig. 6(a), 90 degree of the phase is 7.89 GHz on four half oval-ring transmission line, which means a resistor placed between the fourth half oval-ring structures will have better isolation at frequency around 7.89 GHz. Moreover, in Fig. 6(b), the lowest point of the |*S*_32_| is between 7 and 8 GHz, which further confirms that the best isolation of |*S*_32_| between the fourth half oval-ring structures corresponds to the phase of 90 degrees from 7 to 8 GHz. Comparing to the |*S*_32_| of the resistor placed between the fifth and sixth half oval-ring structures, the |*S*_32_| of the resistor placed between the fourth half oval-ring structures shows the best isolation as shown in Fig. 6(b). Thus, the fourth half oval-ring structures is decided to install the resistor.

**Figure 7:**
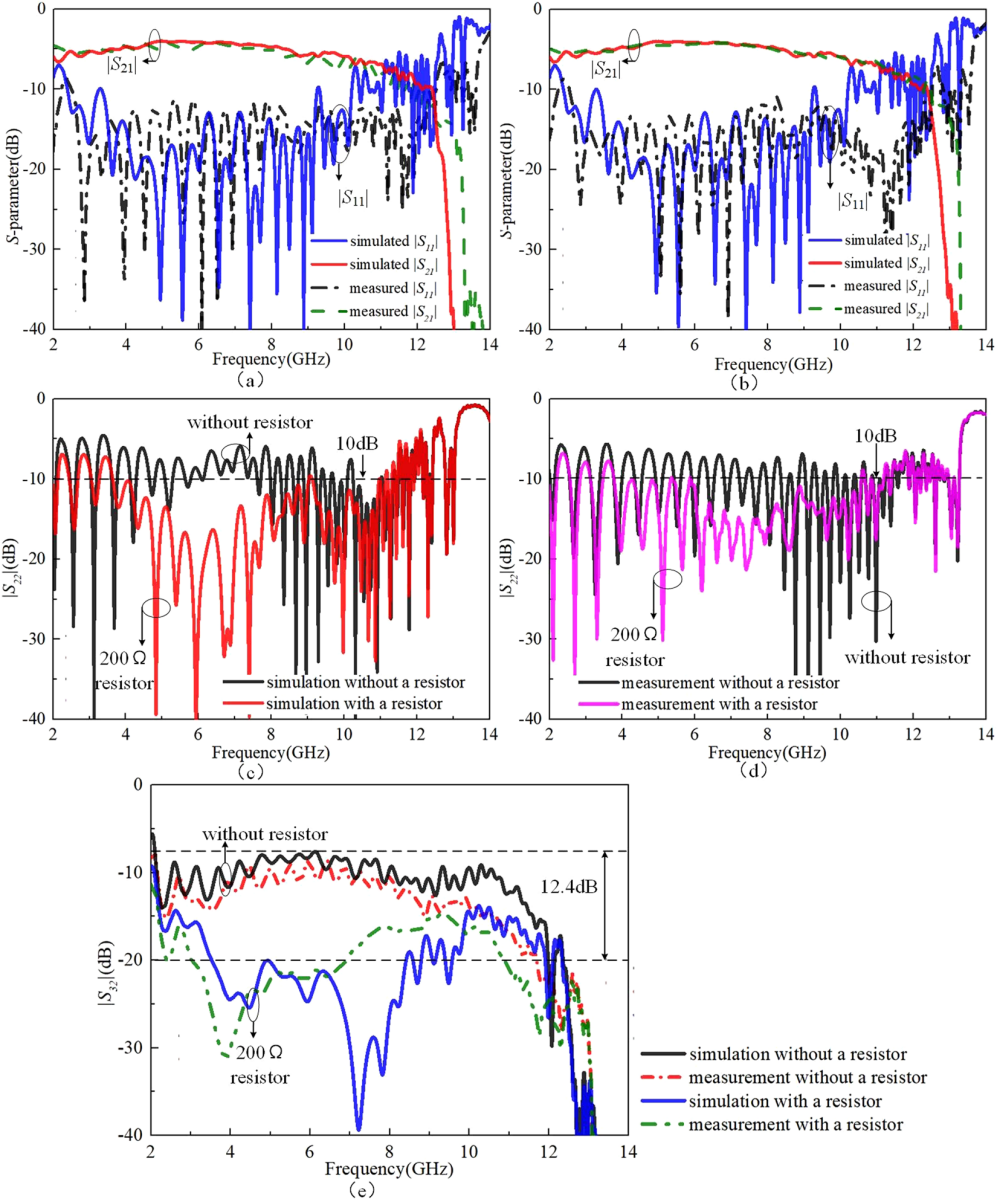
(**a**) |*S*_11_| and |*S*_21_| without a resistor. (**b**) |*S*_11_| and |*S*_21_| with a 200 Ω resistor. (**c**) The comparison of |*S*_22_| with and without a 200 Ω resistor. (**d**) The comparison of |*S*_32_| with and without a 200 Ω resistor.

To demonstrate the expected feature of high-isolation between output channels, a SSPPs power divider adding a single 200 Ω chip resistor is fabricated and measured. Figure 5 shows the photograph of the proposed power divider. Figure 7(a) and (b) present the simulated and measured |*S*_11_|/|*S*_21_| of the power divider without and with a resistor, respectively. Unlike [22], we make a comparison of |*S*_22_| and |*S*_32_| with and without a resistor in Fig. 7(c) and (d) to observe the impact of the resistance on isolation of two outputs. In details, the simulated results are indicated by the solid lines and the measured results are indicated by the dot lines.

As shown in Fig. 7(a) and (b), the simulated |*S*_11_| and |*S*_21_| are similar to each other with limited impact by adding a resistor. The simulated |*S*_11_| is lower than 10 dB from 2 to 10 GHz and the simulated |*S*_21_| is better than 4.5 dB over the frequency range from 4.5 to 7.5 GHz, which confirms the equal power division of the SSPPs wave. In addition, according to Fig. 7(c), |*S*_22_| is worse than 10 dB without a resistor indicating a poor output matching. After adding a resistor, the |*S*_22_| has 6.42 dB improvement over the frequency band 4.5 to 7.5 GHz. Besides, it can be seen that |*S*_32_| with 200 Ω resistor in Fig. 7(d) has port isolation better than 15 dB. Compared to *|S*_32_*|* without a resistor, |*S*_32_| with a resistor obviously has a significant port isolation improvement of 12.4 dB over 4.5 to 7.5 GHz. Based on Fig. 7(c) and (d), the chip resistor significantly improves the impedance matching of the two output ports and provides a good isolation between the ports 2 and 3 in a wide frequency range. The difference between the simulation and measurement results are attributed to the soldering location and the actual volume of the resistor itself, which are not considered in the simulation.

## Methods

### Simulation

As shown in Fig. 1(e–g), we use the commercial software CST Microwave Studio to calculate the electromagnetic field distributions of the designed SSPPs waveguide at 8 GHz. Moreover, the S-parameters of two SSPPs waveguides and power divider are simulated in Figs 2(b), 3(b) and 6(a–d), respectively.

### Fabrication and measurement

The presented structures in Figs 2(a) and 5(d) are fabricated on 0.8 mm thickness dielectric substrate whose dielectric constant and the loss tangent are 2.55 and 0.0029, respectively. On the top of the substrate, there is a copper layer with a thickness of 0.017 mm. In the experiment, the commercial measurement system Agilent N5230A vector network analyzer is used to measure the S-parameters. Two 50 Ω coaxial cables are connected to two 50 Ω SMA connectors at the input/output ports of the designed SSPPs waveguide. We firstly measured the proposed power divider without a resistor and then measured the structure by adding a 200 Ω chip resistor, YAGEO 0201 FR-07. Two tested ports are connected to the coaxial cables, meanwhile the remaining port is terminated by a 50 Ω matching load. The simulations and measurements are in good agreement, which verifies the predicted performance of the designed SSPPs waveguide and power divider.

## Conclusion

A wideband SSPPs waveguide consisting of oval-ring structures is proposed in this work. The designed matching section with ten oval-rings in the gradient scale and flaring ground is further simplified to achieve the high-efficiency conversion between the SSPPs waveguide and the CPWs with 50 Ω characteristic impedance. Based on the SSPPs waveguide, a power divider with stepped-impedance matching is presented, in which the energy of the SSPPs wave is equally divided into two parts. Meanwhile, a single 200 Ω resistor is engineered between the two output channels of the power divider. To the author’s best knowledge, this is the first time that a SSPPs inspired power divider with an isolation-resistor is reported. The reported power divider achieves a good isolation between the two output channels with the good impedance matching at the two output ports in broadband frequency. The designed SSPPs power divider with such good output isolation provides an effective solution for the development of the advanced SSPPs devices and circuits.
